# Supplementary material for: The Healthy Brain 9 (HB9): A new instrument to characterize subjective cognitive decline, and detect anosognosia in mild cognitive impairment
Source: PLoS One. 2025 Jun 24;20(6):e0322351. doi: 10.1371/journal.pone.0322351 (PMC12186938; doi:10.1371/journal.pone.0322351)
Supplement: Attachment — Submitted filename: REVIEW_ A new measure of subjective cognitive complaints.pdf [file pone.0322351.s001.pdf]

Thank you for the opportunity to review this manuscript. It tackles the important and clinically relevant areas of subjective cognitive decline (SCD) and anosognosia in mild cognitive impairment (MCI) by introducing the novel Healthy Brain 9 (HB9) instrument. This is certainly an original contribution, and the initial findings show promise. However, I believe significant revisions are needed to fully realize the potential of this work, particularly regarding the methodology and the interpretation of the results.

Looking at the **Introduction**, the research question is well-defined and addresses a clear gap in the field. The literature review is reasonably comprehensive and current. To make the manuscript's foundation even stronger, I'd suggest expanding the background discussion to more explicitly compare the HB9 with other existing, validated tools for SCD and anosognosia. Showing precisely how the HB9 overcomes limitations or fills specific niches left by current instruments would strengthen the rationale. Mentioning relevant reporting standards, like the COSMIN guidelines for patient-reported outcomes, early on might also help frame the psychometric approach more effectively.

Turning to the **Methodology**, the study utilizes a sample size and diversity that appear adequate for good external validity, and the documentation of recruitment and ethical procedures (IRB approval, consent) is thorough. The choice of statistical analyses like Cronbach's alpha, ROC curves, regression, and ANOVA is generally appropriate for the initial validation goals. However, a key limitation is the cross-sectional design. While suitable for a first look, it inherently prevents drawing conclusions about causality or the progression of SCD/anosognosia over time. This should be clearly acknowledged, perhaps with stronger recommendations for future longitudinal studies. Another point needing careful attention is the potential for recall bias due to the 5-year retrospective self-report period. While acknowledged, the manuscript could delve deeper into this limitation and ideally suggest concrete ways to mitigate it in future research, such as using ecological momentary assessment (EMA). There's also a critical discrepancy concerning the QDRS cut-off score – the text mentions ≥2, while Table 1 seems to use a mean of 1.6. This inconsistency needs immediate clarification and correction to ensure the methodology is reproducible and sound. Finally, while the statistical methods chosen are standard, the reported moderate sensitivity in the ROC analyses raises questions about the HB9's clinical utility for detecting subtle cognitive changes. It might be beneficial to explore more advanced machine learning techniques (like random forests or gradient boosting) with cross-validation, which could potentially improve diagnostic accuracy. Using specialized software like R (with packages such as "mirt" or "lavaan") or Python could also add a layer of analytical rigor and reproducibility.

In the **Results** section, the findings are generally presented clearly, and the tables are helpful for understanding the psychometric properties, group comparisons, and ROC analyses. However, the interpretation could go further by explicitly discussing

the *clinical significance* of the findings, especially the implications of the moderate sensitivity observed in the ROC curves. What does this level of sensitivity mean practically for clinicians? The relatively weak correlations found between HB9 scores and objective cognitive tests or biomarkers also warrant more critical examination. What might this suggest about the construct the HB9 is measuring? Is it capturing subjective concerns distinct from objective performance? Furthermore, the influence of education seems somewhat underexplored. Simply noting a modest correlation isn't quite enough; conducting subgroup analyses or using methods like structural equation modeling (SEM) to probe how education moderates HB9 scores would provide valuable insights.

The **Discussion** does a good job of placing the findings within the context of existing literature and outlining potential clinical implications. The authors also effectively acknowledge limitations like the cross-sectional design and educational skew in the sample. However, the discussion would be strengthened by a deeper exploration of the potential impact of recall bias and the practical consequences of the moderate diagnostic sensitivity. When recommending future research, being more explicit about proposing longitudinal designs, EMA, advanced statistical methods, and perhaps psychometric refinements using Item Response Theory (IRT) or Confirmatory Factor Analysis (CFA) would provide a clearer roadmap. Referencing how the validation efforts align, or could better align, with standards like COSMIN would also add weight to the psychometric discussion.

Overall, the manuscript is logically structured, and the figures and tables are clear. Adherence to ethical standards seems good, although adding a specific statement about potential conflicts of interest or intellectual property related to the HB9 instrument itself would enhance transparency.
